# Supplementary material for: Effect of tourniquet use on the risk of revision in total knee replacement surgery: an analysis of the National Joint Registry Data Set
Source: BMJ Open. 2021 Jun 9;11(6):e045353. doi: 10.1136/bmjopen-2020-045353 (PMC8191625; doi:10.1136/bmjopen-2020-045353)
Supplement: Author's manuscript [file bmjopen-2020-045353.draft_revisions.pdf]

BMJ Open is committed to open peer review. As part of this commitment we make the peer review history of every article we publish publicly available.

When an article is published we post the peer reviewers' comments and the authors' responses online. We also post the versions of the paper that were used during peer review. These are the versions that the peer review comments apply to.

The versions of the paper that follow are the versions that were submitted during the peer review process. They are not the versions of record or the final published versions. They should not be cited or distributed as the published version of this manuscript.

BMJ Open is an open access journal and the full, final, typeset and author-corrected version of record of the manuscript is available on our site with no access controls, subscription charges or pay-per-view fees (http://bmjopen.bmj.com).

If you have any questions on BMJ Open's open peer review process please email info.bmjopen@bmj.com

BMJ Open

The effect of tourniquet use on the risk of revision in total knee replacement surgery: an analysis of the National Joint Registry dataset

Journal:	*BMJ Open*
Manuscript ID	bmjopen-2020-045353
Article Type:	Original research
Date Submitted by the Author:	28-Sep-2020
Complete List of Authors:	Farhan-Alanie, Muhamed; University of Warwick Warwick Medical School, Clinical Trials Unit; University Hospitals Coventry and Warwickshire NHS Trust, Trauma and Orthopaedics<br>Lee, Yujin; University of Warwick Warwick Medical School, Clinical Trials Unit<br>Underwood, Martin; University of Warwick Warwick Medical School, Clinical Trials Unit<br>Metcalfe, Andrew; University of Warwick Warwick Medical School, Warwick Clinical Trials Unit; University Hospitals Coventry and Warwickshire NHS Trust, Trauma and Orthopaedics<br>Wilkinson, J. Mark; Sheffield Teaching Hospitals NHS Foundation Trust; The University of Sheffield Department of Oncology and Metabolism<br>Price, Andrew; University of Oxford, Nuffield Department of Orthopaedics, Rheumatology and Musculoskeletal Sciences<br>Warwick, Jane; University of Warwick Warwick Medical School, Warwick Clinical Trials Unit<br>Wall, Peter ; University of Warwick Warwick Medical School, Warwick Clinical Trials Unit; University Hospitals Coventry and Warwickshire NHS Trust, Trauma and Orthopaedics
Keywords:	Adult orthopaedics < ORTHOPAEDIC & TRAUMA SURGERY, Knee < ORTHOPAEDIC & TRAUMA SURGERY, Limb reconstruction < ORTHOPAEDIC & TRAUMA SURGERY, Orthopaedic & trauma surgery < SURGERY

SCHOLARONE™
Manuscripts

BMJ

I, the Submitting Author has the right to grant and does grant on behalf of all authors of the Work (as defined in the below author licence), an exclusive licence and/or a non-exclusive licence for contributions from authors who are: i) UK Crown employees; ii) where BMJ has agreed a CC-BY licence shall apply, and/or iii) in accordance with the terms applicable for US Federal Government officers or employees acting as part of their official duties; on a worldwide, perpetual, irrevocable, royalty-free basis to BMJ Publishing Group Ltd ("BMJ") its licensees and where the relevant Journal is co-owned by BMJ to the co-owners of the Journal, to publish the Work in this journal and any other BMJ products and to exploit all rights, as set out in our licence.

The Submitting Author accepts and understands that any supply made under these terms is made by BMJ to the Submitting Author unless you are acting as an employee on behalf of your employer or a postgraduate student of an affiliated institution which is paying any applicable article publishing charge ("APC") for Open Access articles. Where the Submitting Author wishes to make the Work available on an Open Access basis (and intends to pay the relevant APC), the terms of reuse of such Open Access shall be governed by a Creative Commons licence – details of these licences and which Creative Commons licence will apply to this Work are set out in our licence referred to above.

Other than as permitted in any relevant BMJ Author's Self Archiving Policies, I confirm this Work has not been accepted for publication elsewhere, is not being considered for publication elsewhere and does not duplicate material already published. I confirm all authors consent to publication of this Work and authorise the granting of this licence.

For peer review only

Title

The effect of tourniquet use on the risk of revision in total knee replacement surgery: an analysis of the National Joint Registry dataset

Authors

Farhan-Alanie MM [1 2], Lee Y [1], Underwood M [1], Metcalfe A[1 2], Wilkinson JM [3], Price A [4], Warwick J [1], Wall PDH [1 2]

1. Warwick Clinical Trials Unit, University of Warwick, Coventry, CV4 7AL, UK.

2. Trauma and Orthopaedics, University Hospitals Coventry and Warwickshire NHS Trust, Coventry, CV2 2DX, UK.

3. Department of Oncology and Metabolism, Department of Oncology and Metabolism, University of Sheffield, University of Sheffield, S10 2RX, UK.

4. Nuffield Department of Orthopaedics, Rheumatology and Musculoskeletal Sciences, University of Oxford, Oxford, OX3 7LD, UK.

Muhamed M Farhan-Alanie – Academic Clinical Fellow Specialty Trainee in Trauma and Orthopaedic Surgery, University of Warwick. muhamed.farhan-alanie@nhs.net (corresponding author). Mobile: 07503237036

Yujin Lee – Postgraduate Research Student, Warwick Clinical Trials Unit. yujin.lee.1@warwick.ac.uk

Martin Underwood – Professor of Primary Care Research, Warwick Clinical Trials Unit. M.Underwood@warwick.ac.uk

Andrew Metcalfe – Associate Professor of Trauma & Orthopaedics, Warwick Clinical Trials Unit. A.Metcalfe@warwick.ac.uk

For peer review only

Jeremy Mark Wilkinson – Professor of Orthopaedic Surgery, University of Sheffield

j.m.wilkinson@sheffield.ac.uk

Andrew Price – Professor of Orthopaedic Surgery, University of Oxford

Andrew.Price@ndorms.ox.ac.uk

Jane Warwick – Head of Statistics, Warwick Clinical Trials Unit J.Warwick@warwick.ac.uk

Peter David Henry Wall – Associate Professor of Trauma & Orthopaedics, Warwick Clinical Trials Unit p.d.h.wall@warwick.ac.uk

Article Summary

Strengths and limitations of this study

- This is the largest and longest cohort comparing revision for all-causes following TKR surgery performed with versus without a tourniquet
- Approximately 17,000 TKR procedures from over 384 centres were included in this study enabling generalisability of our results to similar patient groups
- We have considered all-cause revision as our primary outcome to mitigate the risk of misclassification of indication for revision
- Patients were followed up for a median of 12.2 years from their primary procedure and we have accounted for patient death in our analysis
- We could not control for confounding factors including tourniquet inflation timing, pressure and duration as these details were not captured/collected in the NJR minimum dataset and patients were not randomised.

Background

For peer review only - http://bmjopen.bmj.com/site/about/guidelines.xhtml

Total knee replacement (TKR) is widely used to relieve pain and improve function for individuals with end-stage symptomatic arthritis of the knee.[1,2] In the United Kingdom (UK), the majority of TKR components are cemented in place to hold and stabilise them in the correct position in the bone.[3] The surgery can be performed with or without the use of a thigh tourniquet. Survey research has identified that most surgeons in the UK and around the world perform TKR with the aid of a tourniquet and that this practice has remained unchanged over time.[4-6] In 2003, 93% of primary TKRs were performed with a tourniquet. A 2015 a survey of the British Association of Knee Surgeons (BASK) indicated that 90% of surgeons routinely use a tourniquet.[7,8] The reasons for the widespread use of a tourniquet include a belief that bleeding bone surfaces might impair the fixation of cemented prostheses and reduce long-term implant survivorship.[7] There are data from laboratory animal studies that suggest blood interferes with the strength of the bone-cement interface.[9] This may translate to an increased risk of aseptic loosening of the implant components, the most common cause of TKR failure necessitating revision surgery.[3] However, there is also evidence suggesting that tourniquets do not improve implant fixation and longevity. Two randomised controlled trials (RCT) in humans which used radiostereometric analysis (RSA – a surrogate marker of long term implant survival) demonstrated no significant differences in tibial component migration between TKRs performed with versus without tourniquet at up to two years follow up.[10,11]

Tourniquet use is not without its risks and has been associated with various adverse effects such as increased pain and venous thromboembolism.[12] They have also been associated with other complications including haematoma formation, wound discharge, skin injuries and nerve palsy as well as reduced knee range of motion, even two years after surgery.[4,10] The routine use of tourniquets in TKR surgery therefore requires scrutiny to determine whether they help to reduce the risk of revision surgery or merely expose patients to unnecessary harm. This study compares the all-cause revision rate between primary cemented total knee replacement (TKR) performed with or

For peer review only - http://bmjopen.bmj.com/site/about/guidelines.xhtml

without tourniquet. If tourniquet use is effective in improving the bone-cement interface then lower rates of revision would be observed in the tourniquet group.

Methods

This work forms part of a larger research project titled Safety and Feasibility Evaluation of Tourniquets in Knee Replacement Surgery (SAFE-TKR) and adhered to the published protocol.[13] This work was funded by the National Institute for Health Research (NIHR) Post-Doctoral Fellowship Award (XXXXXXBLINDEDXXXXXXXXXX).

The project was approved by the National Joint Registry (NJR) Research Committee (May 2016) and National Research Ethics Committee (January 2016) (15/WM/0455). Data covering England and Wales were provided by the NJR for the period in which tourniquet use was recorded as part of the minimum dataset for knee replacement surgery (April 2003 to December 2003).[3,8] We analysed only data from primary elective cemented TKRs of which the vast majority were performed for osteoarthritis.[8] Tourniquet use along with other routinely collected NJR baseline variables including age at time of surgery, sex and American Society of Anaesthesiologists (ASA) classification were analysed to measure independent effects, if any, on the rate and timing of all cause revision.

Kaplan-Meier plots were used to compare time to revision between groups which underwent TKR with and without a tourniquet. Cox regression was performed to quantify the difference between the two groups using hazard ratios and to assess the effect of the selected baseline variables on risk of all-cause revision. Time to revision was measured from date of index surgery to the earlier of revision and either date of last follow up or death. All outcomes other than revision were censored. Statistical analyses was performed using Stata 14 – Data and Analysis Software (StataCorp).

For peer review only - http://bmjopen.bmj.com/site/about/guidelines.xhtml

Results

We analysed 20,479 primary knee replacement surgery cases done between April 2003 to December 2003. Of the 406 hospitals listed within the NJR system, 384 had returned data (94%).[5] The numbers undergoing each type of knee replacement surgery, by tourniquet use, are given in Table 1.

Table 1: Types of knee replacement surgery in the England and Wales NJR dataset (April 2003 to December 2003)

	Tourniquet used (N=19,330)	Tourniquet not used (N=1,149)	Total (N=20,479)
**Type of replacement implant, N (%)**			
TKR cemented	16,132 (83%)	842 (73%)	16,974 (83%)
TKR uncemented	1,238 (6%)	228 (20%)	1,466 (7%)
TKR unclassified (e.g. hybrid)	272 (1%)	12 (1%)	284 (1%)
Unicondylar	1,491 (8%)	57 (5%)	1,548 (8%)
Patellofemoral	197 (1%)	10 (1%)	207 (1%)

Our analysis is of the 16,974 cases of primary cemented TKR. Median follow up was 12.2 years (interquartile range 8.4 – 12.6). The baseline characteristics and outcome variables for this subset are shown in Tables 2 and 3, respectively.

For peer review only - http://bmjopen.bmj.com/site/about/guidelines.xhtml

Table 2: Baseline characteristics for all primary cemented TKRs

SD, standard deviation

	**Tourniquet used (N=16,132)**	**Tourniquet not used (N=842)**	**Total (N=16,974)**
Age at Primary Surgery in years, mean (SD)	71 (9)	72 (9)	71 (9)
Patient Gender, N (%)			
Male	6,871 (43%)	382 (45%)	7,253 (43%)
Female	9,261 (57%)	460 (55%)	9,721 (57%)
Primary ASA, N (%)			
P1 – Fit and healthy	4,421 (28%)	235 (28%)	4,656 (28%)
P2 - Mild disease not incapacitating	9,848 (61%)	508 (60%)	10,356 (61%)
P3 - Incapacitating systemic disease	1,828 (11%)	97 (12%)	1,925 (11%)
P4 - Life threatening disease	35 (<1%)	2 (<1%)	37 (<1%)

For peer review only

Table 3: Outcome variables for all primary cemented TKRs

IQR, interquartile range

For peer review only

	Tourniquet used (N=16,132)	Tourniquet not used (N=842)	Total (N=16,974)
Outcome, N (%)			
Unrevised	9,490 (59%)	466 (55%)	9,956 (59%)
Revised	493 (3%)	30 (4%)	523 (3%)
Unrevised at time of death	6,149 (38%)	346 (41%)	6,495 (38%)
Time to outcome in years, median (IQR)	12.2 (8.4 – 12.6)	12.2 (7.5 – 12.6)	12.2 (8.4 – 12.6)
Timing of death, N (%)			
Within 30 days	59 (<1%)	3 (<1%)	62 (<1%)
31 – 90 days	41 (<1%)	3 (<1%)	44 (<1%)
After 90 days	5,946 (37%)	443 (53%)	6,389 (38%)

Figure 1 depicts the Kaplan-Meier survival curves for time to all cause revision in patients having TKR surgery with and without a tourniquet.

Hazard ratios and associated 95% confidence intervals from the univariable and multivariable Cox regression models are shown in Table 4. In the univariable model, there was no evidence that the rate

For peer review only - http://bmjopen.bmj.com/site/about/guidelines.xhtml

at which revisions occur over the study period of 13.3 years differs between those undergoing surgery with a tourniquet and those undergoing surgery without (HR=0.83, 95% CI 0.57 – 1.20, p = 0.31). Of the selected variables considered, only age at primary surgery and gender were significant in the univariable models. Males were 21% more likely than females to undergo revision (HR=1.21; 95% CI 1.01 – 1.43, p = 0.03). The relative risk of revision reduced markedly with increasing age at primary surgery (HR (65-69 years vs < 65 years)=0.75, 95% CI 0.60 – 0.94; HR (70-79 years vs < 65 years)=0.41, 95%CI 0.33-0.50), HR (>80 years vs < 65 years)=0.18, 95% CI 0.11-0.27), p<0.001 (global test)). After adjusting for age at primary surgery, gender and primary ASA grade in the multivariable model, the effect of tourniquet use on the rate at which revisions occur remained largely unchanged (HR=0.82; 95% CI 0.57 – 1.18, p = 0.29). Age at primary surgery remained highly significant in the multivariable model and the hazard ratios did not change (HR (65-69 years vs < 65 years)=0.74, 95% CI 0.60 – 0.92; HR (70-79 years vs < 65 years)=0.40, 95%CI 0.32-0.49), HR (>80 years vs < 65 years)=0.17, 95% CI 0.11-0.27), p<0.001 (global test)) suggesting that age is both a strong and independent predictor of risk of all cause revision.

The difference in percentage unrevised over time (cemented TKR with tourniquet minus cemented TKR without a tourniquet) is presented, together with pointwise confidence intervals, in Figure 2. At 5 years, 2.1% had undergone revision (95% CI 1.3% – 3.3%) in the no tourniquet group and 1.7% in the tourniquet group (95% CI 1.49% – 1.9%). At 10 years, 3.8% had undergone revision (95% CI 2.6% - 5.5%) in the no tourniquet group compared to 3.1% in the tourniquet group (95% CI 2.8% - 3.4%). Reference lines (indicated on Figure 2 in red) indicate where the magnitude of this difference exceeds 1%. The point estimates of the difference (solid blue line) lie within these two limits throughout. The upper end of the confidence interval does, however, exceed this limit from approximately 3 years onwards. Thus, a difference of more than 1% in either direction between the tourniquet and no tourniquet groups cannot be ruled out.

For peer review only - http://bmjopen.bmj.com/site/about/guidelines.xhtml

For peer review only

Table 4: Hazard ratios and associated 95% confidence interval from the univariable and multivariable Cox regression models for all cause revision

	Univariable models		Multivariable model	
	**Hazard ratio (95% CI)**	**p value**	**Hazard ratio (95% CI)**	**p value**
Tourniquet used				
No	1.00		1.00	
Yes	0.83 (0.57 – 1.20)	0.31	0.82 (0.57 – 1.18)	0.29
Age at Primary Surgery				
< 65 years	1.00		1.00	
65-69 years	0.75 (0.60 – 0.94)		0.74 (0.60 – 0.93)	
70-79 years	0.41 (0.33 – 0.50)		0.40 (0.32 – 0.49)	
> 80 years	0.18 (0.11 – 0.27)	<0.001	0.17 (0.11 – 0.27)	<0.001
Patient Gender				
Female	1.00		1.0	
Male	1.21 (1.01– 1.43)	0.03	1.12 (0.94 – 1.33)	0.21
Primary ASA (N, %)				
P1	1.00		1.00	
P2	0.90 (0.74 – 1.10)		1.08 (0.89 – 1.31)	
P3	1.13 (0.84 – 1.51)		1.39 (1.04 – 1.87)	
P4	1.34 (0.19 – 9.54)	0.39	1.61 (0.22 – 11.48)	0.19

For peer review only - http://bmjopen.bmj.com/site/about/guidelines.xhtml

For peer review only

Discussion

This analysis used data from the world's largest TKR audit dataset, the NJR, and enabled us to examine the association of tourniquet use with all-cause revision surgery following primary, elective cemented TKR. Our results show similar revision rates between people undergoing primary TKR with versus without tourniquet up to 13.3 years after surgery. The only baseline variable studied that showed evidence of an independent effect on the risk of revision was age at primary surgery, consistent with previous research.[14] We also observed that a tourniquet was used in 95% of cemented TKRs and 84% of cementless TKRs. From this, we infer that tourniquet use might be greater in cemented procedures because of a perceived association with better cementation quality. However, this association may have arisen by chance.

Our findings indicate that performing TKR with a tourniquet is not associated with any clinically meaningful difference in the risk of revision compared to surgery without a tourniquet. These findings are in keeping with two RCTs using radiostereometric analysis as an outcome which found no significant differences in tibial component migration between TKRs performed with versus without tourniquet at up to two years follow up.[10,11]

A Cochrane systematic review has shown that using a tourniquet for TKR surgery is associated with an increased risk of serious adverse events which includes venous thromboembolism, infection, reoperation and mortality.[12] Other complications including blisters, haematoma, wound oozing, bruising and nerve palsy have also been shown to be more common with tourniquet use during TKR surgery and the devices themselves are frequently colonised with bacteria.[4,15,16] It has also shown that using a tourniquet has no overall effect on total blood loss following surgery.[4] It is perhaps surprising that in the absence of any high quality evidence demonstrating any benefits from using a tourniquet that they continue to have such widespread use. Furthermore, there may be a risk of systemic emboli

For peer review only - http://bmjopen.bmj.com/site/about/guidelines.xhtml

occurring following the deflation of a tourniquet.[17] Transcranial doppler ultrasound studies have found echogenic material in the Circle of Willis after a tourniquet is released and that micro-embolism can occur even in the absence of a patent foramen ovale.[18] The benefits of tourniquet use during TKR surgery must therefore be balanced against the risks.

We analysed approximately 17,000 TKR procedures from over 384 centres which represents 94% of hospitals listed in the NJR at that time.[8] Patients were also followed up for a median of 12.2 years from their primary procedure enabling any potential differences in revision rates to become more apparent particularly as risk of revision for aseptic loosening increases over time.[3] We have used all-cause revision as the endpoint in our study to mitigate risk of misclassification of aseptic loosening within the NJR. This is a global outcome that considers other failure mechanisms responsible for revision procedure such as prosthetic joint infection, especially considering that wound complications have been associated with tourniquet usage.[4,19] This association is believed to be due to a combination of factors including wound ischaemia and local inflammation.[20,21] The relatively short duration in which tourniquet data was captured within the NJR minimum dataset (April to December 2003) reduces biases by time-dependent unknown variables, such as cementation techniques and polyethylene modification, but does limit the sample size. Conversely, the minimum dataset has evolved over time to include established risk factors for revision such as Body Mass Index which were not captured within version 1 and therefore could not be included in our regression analysis.[22,23] Further limitations also related to application of registry based data includes missing data and under reporting of procedures preventing the linkage of revision procedures to any index procedure.[3,24] We acknowledge that we did not consider surgery-related factors such as implant brand, constraint and bearing type in our analysis. This was due to very limited numbers in these subgroups which would not add further meaning to our analysis. Cement brand and viscosity were also not considered however antibiotic content has not been shown to affect all-cause revision.[25] However, we have collected data that encompasses the diversity of implant designs and cement characteristics across routine practice in the UK, and this helps ensure wide generalisability of the results.

For peer review only - http://bmjopen.bmj.com/site/about/guidelines.xhtml

Due to the defined minimum data set, we could not account for other factors such as use of tranexamic acid and application of hypotensive anaesthesia which are used to limit bleeding from cancellous bone ends in an attempt to improve the bone-cement interface. Also, variability in tourniquet inflation timing, pressures and duration introduces heterogeneity within the tourniquet group with some surgeons maintaining inflation throughout the entire procedure and others only during the cementation process. The study is however reflective of real-life routine. Lastly, there is also a risk of bias in our cohort study due to confounding by indication, however an RCT to address this is not feasible given the number of participants required to capture small differences in implant survival and the extended follow up required. Despite the majority of participants (94.4%) having surgery with a tourniquet, there were a large number in the no tourniquet group, and the baseline patient characteristics between the two groups are broadly similar.

Conclusions

Our study has shown a similar risk of revision for primary cemented TKRs performed with and without a tourniquet. However, we are unable to exclude conclusively a clinically important improvement in long term survival for cemented TKRs undertaken with a tourniquet. Surgeons should carefully consider these study's findings as well as the known risks and benefits of tourniquet use, and discuss these issues during the consent process to allow patients to make an informed decision.

Acknowledgements

XXXXXXBLINDEDXXXXXXXXXX

Word count: 2,464 words

For peer review only - http://bmjopen.bmj.com/site/about/guidelines.xhtml

For peer review only

References

1. Wood AM, Brock TM, Heil K, Holmes R, Weusten A. A Review on the Management of Hip and Knee Osteoarthritis. Int J Chronic Dis. 2013;2013:845015.
2. Bourne RB, Chesworth B, Davis A, Mahomed N, Charron K. Comparing patient outcomes after THA and TKA: is there a difference? Clin Orthop Relat Res. 2010;468(2):542-6.
3. NJR Steering Committee. National Joint Registry for England, Wales, Northern Ireland and the Isle of Man: 16th annual report. Hemel Hempstead: National Joint Registry Centre, 2019.
4. Zhang W, Li N, Chen S, Tan Y, Al-Aidaros M, Chen L. The effects of a tourniquet used in total knee arthroplasty: a meta-analysis. J Orthop Surg Res. 2014;9(1):13.
5. National Joint Registry for England and Wales. National Joint Registry for England and Wales 1st Annual Report 2004.
6. Abdel MP, Berry DJ. Current Practice Trends in Primary Hip and Knee Arthroplasties Among Members of the American Association of Hip and Knee Surgeons: A Long-Term Update. J Arthroplasty. 2019;34(7S):S24-S7.
7. Gibbs V, Price A, Wall P, Group S-TS. Surgical tourniquet use in total knee replacement surgery: a survery of BASK members. Knee. 2016;23(4):III-IV.
8. NJR Steering Committee. National Joint Registry for England, Wales, Northern Ireland and the Isle of Man: 1st annual report. Hemel Hempstead: National Joint Registry Centre, 2004.
9. Bannister GC, Miles AW. The influence of cementing technique and blood on the strength of the bone-cement interface. Eng Med. 1988;17(3):131-3.
10. Ledin H, Aspenberg P, Good L. Tourniquet use in total knee replacement does not improve fixation, but appears to reduce final range of motion. Acta Orthop. 2012;83(5):499-503.
11. Molt M, Harsten A, Toksvig-Larsen S. The effect of tourniquet use on fixation quality in cemented total knee arthroplasty a prospective randomized clinical controlled RSA trial. Knee. 2014;21(2):396-401.
12. Ahmed I, Chawla A, Underwood M, Price A, Metcalfe A, Hutchinson C, et al. Tourniquet use for knee replacement surgery. Cochrane Database of Systematic Reviews. In Press.
13. Wall PD, Ahmed I, Metcalfe A, Price AJ, Seers K, Hutchinson CE, et al. Safety and feasibility evaluation of tourniquets for total knee replacement (SAFE-TKR): study protocol. BMJ Open. 2018;8(4):e022067.
14. Bayliss LE, Culliford D, Monk AP, Glyn-Jones S, Prieto-Alhambra D, Judge A, et al. The effect of patient age at intervention on risk of implant revision after total replacement of the hip or knee: a population-based cohort study. Lancet. 2017;389(10077):1424-30.
15. Alcelik I, Pollock RD, Sukeik M, Bettany-Saltikov J, Armstrong PM, Fismer P. A comparison of outcomes with and without a tourniquet in total knee arthroplasty: a systematic review and meta-analysis of randomized controlled trials. J Arthroplasty. 2012;27(3):331-40.
16. Ahmed SM, Ahmad R, Case R, Spencer RF. A study of microbial colonisation of orthopaedic tourniquets. Ann R Coll Surg Engl. 2009;91(2):131-4.
17. Berman AT, Parmet JL, Harding SP, Israelite CL, Chandrasekaran K, Horrow JC, et al. Emboli observed with use of transesophageal echocardiography immediately after tourniquet release during total knee arthroplasty with cement. The Journal of bone and joint surgery American volume. 1998;80(3):389-96.

For peer review only - http://bmjopen.bmj.com/site/about/guidelines.xhtml

18. Sulek CA, Davies LK, Enneking FK, Gearen PA, Lobato EB. Cerebral microembolism diagnosed by transcranial Doppler during total knee arthroplasty: correlation with transesophageal echocardiography. Anesthesiology. 1999;91(3):672-6.
19. Garbedian S, Sternheim A, Backstein D. Wound healing problems in total knee arthroplasty. Orthopedics. 2011;34(9):e516-8.
20. Clarke MT, Longstaff L, Edwards D, Rushton N. Tourniquet-induced wound hypoxia after total knee replacement. J Bone Joint Surg Br. 2001;83(1):40-4.
21. Carden DL, Granger DN. Pathophysiology of ischaemia-reperfusion injury. J Pathol. 2000;190(3):255-66.
22. Namba RS, Cafri G, Khatod M, Inacio MC, Brox TW, Paxton EW. Risk factors for total knee arthroplasty aseptic revision. J Arthroplasty. 2013;28(8 Suppl):122-7.
23. Kerkhoffs GM, Servien E, Dunn W, Dahm D, Bramer JA, Haverkamp D. The influence of obesity on the complication rate and outcome of total knee arthroplasty: a meta-analysis and systematic literature review. J Bone Joint Surg Am. 2012;94(20):1839-44.
24. Sabah SA, Henckel J, Cook E, Whittaker R, Hothi H, Pappas Y, et al. Validation of primary metal-on-metal hip arthroplasties on the National Joint Registry for England, Wales and Northern Ireland using data from the London Implant Retrieval Centre: a study using the NJR dataset. The bone & joint journal. 2015;97-B(1):10-8.
25. Bohm E, Zhu N, Gu J, de Guia N, Linton C, Anderson T, et al. Does adding antibiotics to cement reduce the need for early revision in total knee arthroplasty? Clin Orthop Relat Res. 2014;472(1):162-8.

For peer review only

For peer review only - http://bmjopen.bmj.com/site/about/guidelines.xhtml

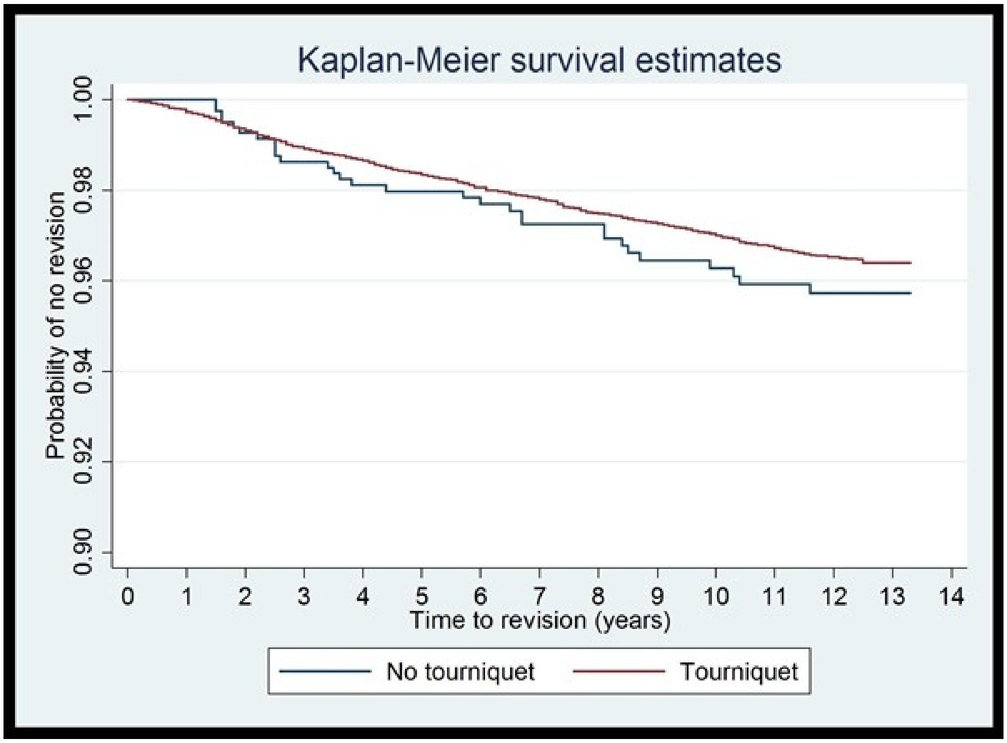

Figure 1: Time to all cause revision by tourniquet use

158x116mm (96 x 96 DPI)

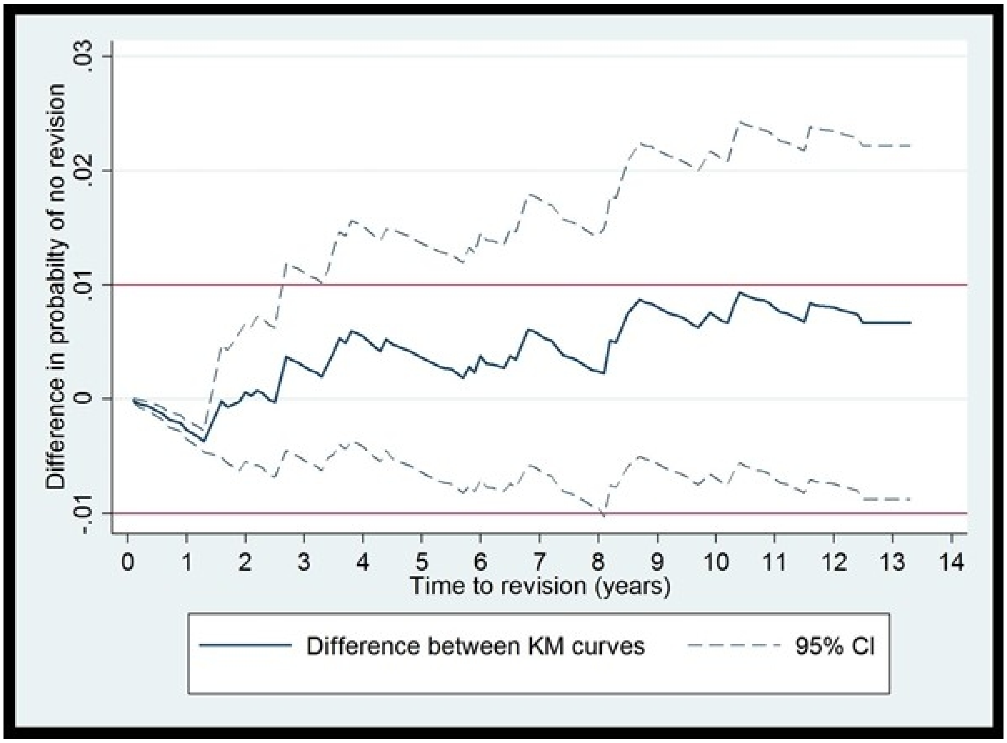

Figure 2: Difference in time to revision (TKR with tourniquet - TKR without tourniquet), over time (unadjusted)

158x116mm (96 x 96 DPI)

BMJ Open

The effect of tourniquet use on the risk of revision in total knee replacement surgery: an analysis of the National Joint Registry dataset

Journal:	*BMJ Open*
Manuscript ID	bmjopen-2020-045353.R1
Article Type:	Original research
Date Submitted by the Author:	16-Mar-2021
Complete List of Authors:	Farhan-Alanie, Muhamed; University of Warwick Warwick Medical School, Clinical Trials Unit; University Hospitals Coventry and Warwickshire NHS Trust, Trauma and Orthopaedics<br>Lee, Yujin; University of Warwick Warwick Medical School, Clinical Trials Unit<br>Underwood, Martin; University of Warwick Warwick Medical School, Clinical Trials Unit<br>Metcalfe, Andrew; University of Warwick Warwick Medical School, Warwick Clinical Trials Unit; University Hospitals Coventry and Warwickshire NHS Trust, Trauma and Orthopaedics<br>Wilkinson, J. Mark; Sheffield Teaching Hospitals NHS Foundation Trust; The University of Sheffield Department of Oncology and Metabolism<br>Price, Andrew; University of Oxford, Nuffield Department of Orthopaedics, Rheumatology and Musculoskeletal Sciences<br>Warwick, Jane; University of Warwick Warwick Medical School, Warwick Clinical Trials Unit<br>Wall, Peter ; University of Warwick Warwick Medical School, Warwick Clinical Trials Unit; University Hospitals Coventry and Warwickshire NHS Trust, Trauma and Orthopaedics
**Primary Subject Heading**:	Surgery
Secondary Subject Heading:	Surgery
Keywords:	Adult orthopaedics < ORTHOPAEDIC & TRAUMA SURGERY, Knee < ORTHOPAEDIC & TRAUMA SURGERY, Orthopaedic & trauma surgery < SURGERY, ORTHOPAEDIC & TRAUMA SURGERY, Limb reconstruction < ORTHOPAEDIC & TRAUMA SURGERY

SCHOLARONE™
Manuscripts

I, the Submitting Author has the right to grant and does grant on behalf of all authors of the Work (as defined in the below author licence), an exclusive licence and/or a non-exclusive licence for contributions from authors who are: i) UK Crown employees; ii) where BMJ has agreed a CC-BY licence shall apply, and/or iii) in accordance with the terms applicable for US Federal Government officers or employees acting as part of their official duties; on a worldwide, perpetual, irrevocable, royalty-free basis to BMJ Publishing Group Ltd ("BMJ") its licensees and where the relevant Journal is co-owned by BMJ to the co-owners of the Journal, to publish the Work in this journal and any other BMJ products and to exploit all rights, as set out in our licence.

The Submitting Author accepts and understands that any supply made under these terms is made by BMJ to the Submitting Author unless you are acting as an employee on behalf of your employer or a postgraduate student of an affiliated institution which is paying any applicable article publishing charge ("APC") for Open Access articles. Where the Submitting Author wishes to make the Work available on an Open Access basis (and intends to pay the relevant APC), the terms of reuse of such Open Access shall be governed by a Creative Commons licence – details of these licences and which Creative Commons licence will apply to this Work are set out in our licence referred to above.

Other than as permitted in any relevant BMJ Author's Self Archiving Policies, I confirm this Work has not been accepted for publication elsewhere, is not being considered for publication elsewhere and does not duplicate material already published. I confirm all authors consent to publication of this Work and authorise the granting of this licence.

For peer review only

Title

The effect of tourniquet use on the risk of revision in total knee replacement surgery: an analysis of the National Joint Registry dataset

Authors

Farhan-Alanie MM [1 2], Lee Y [1], Underwood M [1], Metcalfe A[1 2], Wilkinson JM [3], Price A [4], Warwick J [1], Wall PDH [1 2]

1. Warwick Clinical Trials Unit, University of Warwick, Coventry, CV4 7AL, UK.

2. Trauma and Orthopaedics, University Hospitals Coventry and Warwickshire NHS Trust, Coventry, CV2 2DX, UK.

3. Department of Oncology and Metabolism, Department of Oncology and Metabolism, University of Sheffield, University of Sheffield, S10 2RX, UK.

4. Nuffield Department of Orthopaedics, Rheumatology and Musculoskeletal Sciences, University of Oxford, Oxford, OX3 7LD, UK.

Muhamed M Farhan-Alanie – Academic Clinical Fellow Specialty Trainee in Trauma and Orthopaedic Surgery, University of Warwick. muhamed.farhan-alanie@nhs.net (corresponding author). Mobile: 07503237036

Yujin Lee – Postgraduate Research Student, Warwick Clinical Trials Unit. yujin.lee.1@warwick.ac.uk

Martin Underwood – Professor of Primary Care Research, Warwick Clinical Trials Unit. M.Underwood@warwick.ac.uk

Andrew Metcalfe – Associate Professor of Trauma & Orthopaedics, Warwick Clinical Trials Unit. A.Metcalfe@warwick.ac.uk

For peer review only - http://bmjopen.bmj.com/site/about/guidelines.xhtml

Jeremy Mark Wilkinson – Professor of Orthopaedic Surgery, University of Sheffield

j.m.wilkinson@sheffield.ac.uk

Andrew Price – Professor of Orthopaedic Surgery, University of Oxford

Andrew.Price@ndorms.ox.ac.uk

Jane Warwick – Head of Statistics, Warwick Clinical Trials Unit J.Warwick@warwick.ac.uk

Peter David Henry Wall – Associate Professor of Trauma & Orthopaedics, Warwick Clinical Trials Unit p.d.h.wall@warwick.ac.uk

Abstract

Objective: Tourniquet use in total knee replacement (TKR) is believed to improve the bone-cement interface by reducing bleeding, potentially prolonging implant survival. This study aimed to compare the risk of revision for primary cemented TKR performed with or without a tourniquet.

Design: We analysed data from the National Joint Registry (NJR) for all primary cemented TKRs performed in England and Wales between April 2003 to December 2003. Kaplan-Meier plots and Cox regression were used to assess the influence of tourniquet use, age at time of surgery, sex and American Society of Anaesthesiologists classification on risk of revision for all-causes.

Results: Data were available for 16,974 cases of primary cemented TKR, of which 16,132 had surgery with a tourniquet and 842 had surgery without a tourniquet. At 10 years, 3.8% had had revision (95% CI 2.6% – 5.5%) in the no tourniquet group and 3.1% in the tourniquet group (95%CI 2.8% – 3.4%). After adjusting for age at primary surgery, gender and primary ASA score, the hazard ratio for all cause revision for cemented TKR without a tourniquet was 0.82 (95% CI 0.57 – 1.18).

For peer review only - http://bmjopen.bmj.com/site/about/guidelines.xhtml

Conclusions: We did not find evidence that using a tourniquet for primary cemented TKR offers a clinically important or statistically significant reduction in the risk of all-cause revision up to 13 years after surgery. Surgeons should consider this evidence when deciding whether to use a tourniquet use for cemented TKR.

Article Summary

Strengths and limitations of this study

- This is the largest and longest cohort comparing revision for all-causes following TKR surgery performed with versus without a tourniquet
- Approximately 17,000 TKR procedures from 384 centres were included in this study enabling generalisability of our results to similar patient groups
- We have considered all-cause revision as our primary outcome to mitigate the risk of misclassification of indication for revision
- Patients were followed up for a median of 12.2 years from their primary procedure and we have accounted for patient death in our analysis
- We could not control for confounding factors including tourniquet inflation timing, pressure and duration as these details were not captured/collected in the NJR minimum dataset and patients were not randomised.

Background

For peer review only

Total knee replacement (TKR) is widely used to relieve pain and improve function for individuals with end-stage symptomatic arthritis of the knee.[1,2] In the United Kingdom (UK), the majority of TKR components are cemented in place to hold and stabilise them in the correct position in the bone.[3] The surgery can be performed with or without the use of a thigh tourniquet. Survey research has identified that most surgeons in the UK and around the world perform TKR with the aid of a tourniquet and that this practice has remained unchanged over time.[4-6] In 2003, 93% of primary TKRs were performed with a tourniquet. A 2015 survey of the British Association of Knee Surgeons (BASK) indicated that 90% of surgeons routinely use a tourniquet.[7,8] The reasons for the widespread use of a tourniquet include a belief that bleeding bone surfaces might impair the fixation of cemented prostheses and reduce long-term implant survivorship.[7] There are data from laboratory animal studies that suggest blood interferes with the strength of the bone-cement interface.[9] This may translate to an increased risk of aseptic loosening of the implant components, the most common cause of TKR failure necessitating revision surgery.[3] However, there is also evidence suggesting that tourniquets do not improve implant fixation and longevity. Two randomised controlled trials (RCT) in humans which used radiostereometric analysis (RSA – a surrogate marker of long term implant survival) demonstrated no significant differences in tibial component migration between TKRs performed with versus without tourniquet at up to two years follow up.[10,11]

For peer review only

Tourniquet use is not without its risks and has been associated with various adverse effects such as increased pain and venous thromboembolism.[12] They have also been associated with other complications including haematoma formation, wound discharge, skin injuries and nerve palsy as well as reduced knee range of motion, even two years after surgery.[4,10] The routine use of tourniquets in TKR surgery therefore requires scrutiny to determine whether they help to reduce the risk of revision surgery or merely expose patients to unnecessary harm.[12] This study compares the all-cause revision rate between primary cemented total knee replacement (TKR) performed with or

For peer review only - http://bmjopen.bmj.com/site/about/guidelines.xhtml

without tourniquet. If tourniquet use is effective in improving the bone-cement interface then lower rates of revision would be observed in the tourniquet group.

Methods

This work forms part of a larger research project titled Safety and Feasibility Evaluation of Tourniquets in Knee Replacement Surgery (SAFE-TKR) and adhered to the published protocol.[13] This article reports the findings for risk of revision between patients who underwent TKR surgery with versus without a thigh tourniquet. Other outcomes examining potential adverse events and length of hospital will be reported within a separate article. This work was funded by the National Institute for Health Research (NIHR) Post-Doctoral Fellowship Award (PDF-2015-08-108).

The project was approved by the National Joint Registry (NJR) Research Committee (May 2016) and National Research Ethics Committee (January 2016) (15/WM/0455). Data covering England and Wales were provided by the NJR for the period in which tourniquet use was recorded as part of the minimum dataset for knee replacement surgery (April 2003 to December 2003).[3,8] We analysed only data from primary elective cemented TKRs of which the vast majority were performed for osteoarthritis.[8] Tourniquet use along with other routinely collected NJR baseline variables including age at time of surgery, sex and American Society of Anaesthesiologists (ASA) classification were analysed to measure independent effects, if any, on the rate and timing of all cause revision.

Kaplan-Meier plots were used to compare time to revision between groups which underwent TKR with and without a tourniquet. Cox regression was performed to quantify the difference between the two groups using hazard ratios and to assess the effect of the selected baseline variables on risk of all-cause revision. Time to revision was measured from date of index surgery to the earlier of

For peer review only

For peer review only - http://bmjopen.bmj.com/site/about/guidelines.xhtml

revision and either date of last follow up or death. All outcomes other than revision were censored. Statistical analyses was performed using Stata 14 – Data and Analysis Software (StataCorp).

Patient and Public Involvement (PPI)

Thirty patients who have undergone TKR were surveyed and took part in a focus group to explore their experiences and views towards tourniquet use during their surgery. Results revealed that patients believed further research on tourniquet use in TKR was important. Implant longevity was identified as being a particular concern amongst the patients consulted. Patients contributed to the design of this study by helping determine the important outcomes and their timing. The results of this research will be disseminated to study participants through press releases and social media.

Results

We analysed 20,479 primary knee replacement surgery cases done between April 2003 to December 2003. Of the 406 hospitals listed within the NJR system, 384 had returned data (94%).[5] The numbers undergoing each type of knee replacement surgery, by tourniquet use, are given in Table 1.

Table 1: Types of knee replacement surgery in the England and Wales NJR dataset (April 2003 to December 2003)

	**Tourniquet used (N=19,330)**	**Tourniquet not used (N=1,149)**	**Total (N=20,479)**

For peer review only - http://bmjopen.bmj.com/site/about/guidelines.xhtml

Type of replacement implant, N (%)			
TKR cemented	16,132 (83%)	842 (73%)	16,974 (83%)
TKR uncemented	1,238 (6%)	228 (20%)	1,466 (7%)
TKR unclassified (e.g. hybrid)	272 (1%)	12 (1%)	284 (1%)
Unicondylar	1,491 (8%)	57 (5%)	1,548 (8%)
Patellofemoral	197 (1%)	10 (1%)	207 (1%)

Our analysis is of the 16,974 cases of primary cemented TKR. Median follow up was 12.2 years (interquartile range 8.4 – 12.6). The baseline characteristics and outcome variables for this subset are shown in Tables 2 and 3, respectively.

For peer review only

	**Tourniquet used (N=16,132)**	**Tourniquet not used (N=842)**	**Total (N=16,974)**
Age at Primary Surgery in years, mean (SD)	71 (9)	72 (9)	71 (9)
Patient Gender, N (%)			

For peer review only - http://bmjopen.bmj.com/site/about/guidelines.xhtml

Male	6,871 (43%)	382 (45%)	7,253 (43%)
Female	9,261 (57%)	460 (55%)	9,721 (57%)
Primary ASA, N (%)			
P1 – Fit and healthy	4,421 (28%)	235 (28%)	4,656 (28%)
P2 - Mild disease not incapacitating	9,848 (61%)	508 (60%)	10,356 (61%)
P3 - Incapacitating systemic disease	1,828 (11%)	97 (12%)	1,925 (11%)
P4 - Life threatening disease	35 (<1%)	2 (<1%)	37 (<1%)

Table 2: Baseline characteristics for all primary cemented TKRs

SD, standard deviation

Table 3: Outcome variables for all primary cemented TKRs

IQR, interquartile range

	Tourniquet used	Tourniquet not used	Total

For peer review only - http://bmjopen.bmj.com/site/about/guidelines.xhtml

	**(N=16,132)**	**(N=842)**	**(N=16,974)**
Outcome, N (%)			
Unrevised	9,490 (59%)	466 (55%)	9,956 (59%)
Revised	493 (3%)	30 (4%)	523 (3%)
Unrevised at time of death	6,149 (38%)	346 (41%)	6,495 (38%)
Time to outcome in years, median (IQR)	12.2 (8.4 – 12.6)	12.2 (7.5 – 12.6)	12.2 (8.4 – 12.6)
Timing of death, N (%)			
Within 30 days	59 (<1%)	3 (<1%)	62 (<1%)
31 – 90 days	42 (<1%)	2 (<1%)	44 (<1%)
After 90 days	6,048 (37%)	341 (40%)	6,389 (38%)

Hazard ratios and associated 95% confidence intervals from the univariable and multivariable Cox regression models are shown in Table 4. In the univariable model, there was no evidence that the rate at which revisions occur over the study period of 13.3 years differs between those undergoing surgery with a tourniquet and those undergoing surgery without (HR=0.83, 95% CI 0.57 – 1.20, p = 0.31). Of the selected variables considered, only age at primary surgery and gender were significant in the univariable models. . After adjusting for age at primary surgery, gender and primary ASA grade in the multivariable model, the effect of tourniquet use on the rate at which revisions occur remained largely unchanged (HR=0.82; 95% CI 0.57 – 1.18, p = 0.29). Age at primary surgery remained highly significant in the multivariable model and the hazard ratios did not change (global test; p<0.001) suggesting that age is both a strong and independent predictor of risk of all cause revision. Gender and primary ASA grade did not reach statistical significance in the multivariable model suggesting these were not reliable independent predictors for risk of all cause revision. Figure 1 shows Kaplan-Meier survival curves for time to all cause revision in patients having TKR surgery with and without a tourniquet.

For peer review only

For peer review only - http://bmjopen.bmj.com/site/about/guidelines.xhtml

The difference in percentage unrevised over time (cemented TKR with tourniquet minus cemented TKR without a tourniquet) is presented, together with pointwise confidence intervals, in Figure 2. At 5 years, 2.1% had undergone revision (95% CI 1.3% – 3.3%) in the no tourniquet group and 1.7% in the tourniquet group (95% CI 1.49% – 1.9%). At 10 years, 3.8% had undergone revision (95% CI 2.6% - 5.5%) in the no tourniquet group compared to 3.1% in the tourniquet group (95% CI 2.8% - 3.4%). Reference lines (indicated on Figure 2 in red) indicate where the magnitude of this difference exceeds 1%. The point estimates of the difference (solid blue line) lie within these two limits throughout. The upper end of the confidence interval does, however, exceed this limit from approximately 3 years onwards. Thus, a difference of more than 1% in either direction between the tourniquet and no tourniquet groups cannot be ruled out.

Table 4: Hazard ratios and associated 95% confidence interval from the univariable and multivariable Cox regression models for all cause revision

	Univariable models		Multivariable model	
	**Hazard ratio (95% CI)**	**p value**	**Hazard ratio (95% CI)**	**p value**
Tourniquet used				
No	1.00		1.00	
Yes	0.83 (0.57 – 1.20)	0.31	0.82 (0.57 – 1.18)	0.29
Age at Primary Surgery				
< 65 years	1.00		1.00	
65-69 years	0.75 (0.60 – 0.94)		0.74 (0.60 – 0.93)	
70-79 years	0.41 (0.33 – 0.50)		0.40 (0.32 – 0.49)	

For peer review only - http://bmjopen.bmj.com/site/about/guidelines.xhtml

> 80 years	0.18 (0.11 – 0.27)	<0.001	0.17 (0.11 – 0.27)	<0.001
Patient Gender				
Female	1.00		1.0	
Male	1.21 (1.01– 1.43)	0.03	1.12 (0.94 – 1.33)	0.21
Primary ASA (N, %)				
P1	1.00		1.00	
P2	0.90 (0.74 – 1.10)		1.08 (0.89 – 1.31)	
P3	1.13 (0.84 – 1.51)		1.39 (1.04 – 1.87)	
P4	1.34 (0.19 – 9.54)	0.39	1.61 (0.22 – 11.48)	0.19

For peer review only

Discussion

This analysis used data from the world's largest TKR audit dataset, the NJR, and enabled us to examine the association of tourniquet use with all-cause revision surgery following primary, elective cemented TKR. Our results show similar revision rates between people undergoing primary TKR with versus without tourniquet up to 13.3 years after surgery. The only baseline variable studied that showed evidence of an independent effect on the risk of revision was age at primary surgery, consistent with previous research.[14] We also observed that a tourniquet was used in 95% of cemented TKRs and 84% of cementless TKRs. From this, we infer that tourniquet use might be greater in cemented procedures because of a perceived association with better cementation quality. However, this association may have arisen by chance.

Our findings indicate that performing TKR with a tourniquet is not associated with any clinically meaningful difference in the risk of revision compared to surgery without a tourniquet. These findings are in keeping with two RCTs of 50 and 60 patients who underwent primary cemented TKR surgery for osteoarthritis with or without the use of a tourniquet. No significant differences in tibial component migration was observed at up to two years follow up using radiostereometric analysis.[10,11] However, patient loss to follow-up in both these RCTs resulted in inadequate power to detect differences in migration following one year limiting the validity of the study findings to predict future revision for loosening to between five and ten prosthesis years.[15] In contrast, our study has compared all-cause revision up to 13.3 years post-operatively, and is more reflective of real life practice as it does not limit surgeons to a defined surgical technique or other factors such as choice of implant or bone cement.

For peer review only - http://bmjopen.bmj.com/site/about/guidelines.xhtml

A Cochrane systematic review has shown that using a tourniquet for TKR surgery is associated with an increased risk of serious adverse events which includes venous thromboembolism, infection, reoperation and mortality. Other complications including blisters, haematoma, wound oozing, bruising and nerve palsy have also been shown to be more common with tourniquet use during TKR surgery and the devices themselves are frequently colonised with bacteria.[4,16,17] It has been shown that tourniquet use is associated with a modest statistically significant reduction in intraoperative blood loss. This may improve the surgical field of view, however we are not aware of any published literately formally examining this outcome. It is surprising that in the absence of any high quality evidence demonstrating any likely benefits from using a tourniquet and the existing evidence demonstrating their increased risk of harm to patients that they continue to have such widespread use. Furthermore, there may also be a risk of systemic emboli occurring following the deflation of a tourniquet.[18] Transcranial doppler ultrasound studies have found echogenic material in the Circle of Willis after a tourniquet is released and that micro-embolism can occur even in the absence of a patent foramen ovale.[19] The potential benefits of tourniquet use during TKR surgery must therefore be balanced against the risks.

We analysed approximately 17,000 TKR procedures from over 384 centres which represents 94% of hospitals listed in the NJR at that time.[8] Patients were also followed up for a median of 12.2 years from their primary procedure enabling any potential differences in revision rates to become more apparent particularly as risk of revision for aseptic loosening increases over time.[3] We have used all-cause revision as the endpoint in our study to mitigate risk of misclassification of aseptic loosening within the NJR. This is a global outcome that considers other failure mechanisms responsible for revision procedure such as prosthetic joint infection, especially considering that wound complications have been associated with tourniquet usage.[4,20] This association is believed to be due to a combination of factors including wound ischaemia and local inflammation.[21,22] The relatively short duration in which tourniquet data was captured within the NJR minimum dataset (April to December 2003) reduces biases by time-dependent unknown variables, such as cementation techniques and polyethylene

For peer review only - http://bmjopen.bmj.com/site/about/guidelines.xhtml

modification, but does limit the sample size. Conversely, the minimum dataset has evolved over time to include established risk factors for revision such as Body Mass Index which were not captured within version 1 and therefore could not be included in our regression analysis.[23,24] Further limitations also related to application of registry based data includes missing data and under reporting of procedures preventing the linkage of revision procedures to any index procedure.[3,25] Due to missing data in our dataset, we were not able to perform a sub analysis of revision by indication and assess whether rates of periprosthetic patella fractures and secondary patellar resurfacing differed between the two groups. We acknowledge that we did not consider surgery-related factors such as implant brand, constraint and bearing type in our analysis. This was due to very limited numbers in these subgroups which would not add further meaning to our analysis. Cement brand and viscosity were also not considered however antibiotic content has not been shown to affect all-cause revision.[26] However, we have collected data that encompasses the diversity of implant designs and cement characteristics across routine practice in the UK, and this helps ensure wide generalisability of the results. Another limitation is the relatively small sample size of TKR procedures performed without a tourniquet across a large number of centres (approximately 150 centres) which may have influenced our results due to performance bias and confounding by indication.

Due to the defined minimum data set, we could not account for other factors such as use of tranexamic acid and application of hypotensive anaesthesia which are used to limit bleeding from cancellous bone ends in an attempt to improve the bone-cement interface. Also, variability in tourniquet inflation timing, pressures and duration introduces heterogeneity within the tourniquet group with some surgeons maintaining inflation throughout the entire procedure and others only during the cementation process. The study is however reflective of real-life routine. Lastly, there is also a risk of bias in our cohort study due to confounding by indication, however an RCT to address this is not feasible given the number of participants required to capture small differences in implant survival and the extended follow up required. Despite the majority of participants (95%) having

For peer review only - http://bmjopen.bmj.com/site/about/guidelines.xhtml

surgery with a tourniquet, there were a large number in the no tourniquet group, and the baseline patient characteristics between the two groups are broadly similar.

Conclusions

Our study has shown a similar risk of revision for primary cemented TKRs performed with and without a tourniquet. However, we are unable to exclude conclusively a clinically important improvement in long term survival for cemented TKRs undertaken with a tourniquet. Surgeons should carefully consider these study's findings as well as the known risks and benefits of tourniquet use and discuss these issues during the consent process to allow patients to make an informed decision.

Word count: 2,733 words

Acknowledgements

We thank the patients and staff of all the hospitals who have contributed data to the National Joint Registry. We are grateful to the Healthcare Quality Improvement Partnership, the National Joint Registry Steering Committee, and staff at the National Joint Registry for facilitating this work. We would like also like to thank all patients and public members/representatives who were involved in this study and in particular CG, JS and JD for their ongoing contribution throughout the study.

Contributorship statement

MMFA: drafted and reviewed the final manuscript. MU, AM, MJW and AJP: study design, drafted and reviewed the final manuscript. YL and JW: data analysis, drafted and reviewed the final manuscript. PDHW: study conception, study design, data analysis, drafted and reviewed the final manuscript.

Competing interests

For peer review only - http://bmjopen.bmj.com/site/about/guidelines.xhtml

None declared.

Funding

This study presents research funded by a National Institute for Health Research (NIHR) Post-Doctoral Fellowship Award (PDF-2015-08-108).

Data sharing statement

Access to the data analysed in this study required permission from the National Joint Registry for England, Wales and Northern Ireland Research Sub-committee. http://www.njrcentre.org.uk/njrcentre/Research/Researchrequests/tabid/305/Default.aspx contains information on research data access request to the National Joint Registry.

Ethics approval

The project was approved by the National Joint Registry (NJR) Research Committee (May 2016) and National Research Ethics Committee (January 2016) (15/WM/0455). Patient consent was obtained for data collection by the National Joint Registry. According to the specifications of the NHS Health Research Authority, separate informed consent and ethical approval were not required for the present study.

Figure captions

Figure 1: Time to all cause revision by tourniquet use

Figure 2: Difference in time to revision (TKR with tourniquet - TKR without tourniquet), over time (unadjusted)

For peer review only - http://bmjopen.bmj.com/site/about/guidelines.xhtml

For peer review only

References

1. Wood AM, Brock TM, Heil K, Holmes R, Weusten A. A Review on the Management of Hip and Knee Osteoarthritis. Int J Chronic Dis. 2013;2013:845015.
2. Bourne RB, Chesworth B, Davis A, Mahomed N, Charron K. Comparing patient outcomes after THA and TKA: is there a difference? Clin Orthop Relat Res. 2010;468(2):542-6.
3. NJR Steering Committee. National Joint Registry for England, Wales, Northern Ireland and the Isle of Man: 16th annual report. Hemel Hempstead: National Joint Registry Centre, 2019.
4. Zhang W, Li N, Chen S, Tan Y, Al-Aidaros M, Chen L. The effects of a tourniquet used in total knee arthroplasty: a meta-analysis. J Orthop Surg Res. 2014;9(1):13.
5. National Joint Registry for England and Wales. National Joint Registry for England and Wales 1st Annual Report 2004.
6. Abdel MP, Berry DJ. Current Practice Trends in Primary Hip and Knee Arthroplasties Among Members of the American Association of Hip and Knee Surgeons: A Long-Term Update. J Arthroplasty. 2019;34(7S):S24-S7.
7. Gibbs V, Price A, Wall P, Group S-TS. Surgical tourniquet use in total knee replacement surgery: a survery of BASK members. Knee. 2016;23(4):III-IV.
8. NJR Steering Committee. National Joint Registry for England, Wales, Northern Ireland and the Isle of Man: 1st annual report. Hemel Hempstead: National Joint Registry Centre, 2004.
9. Bannister GC, Miles AW. The influence of cementing technique and blood on the strength of the bone-cement interface. Eng Med. 1988;17(3):131-3.
10. Ledin H, Aspenberg P, Good L. Tourniquet use in total knee replacement does not improve fixation, but appears to reduce final range of motion. Acta Orthop. 2012;83(5):499-503.
11. Molt M, Harsten A, Toksvig-Larsen S. The effect of tourniquet use on fixation quality in cemented total knee arthroplasty a prospective randomized clinical controlled RSA trial. Knee. 2014;21(2):396-401.
12. Ahmed I, Chawla A, Underwood M, Price AJ, Metcalfe A, Hutchinson C, et al. Tourniquet use for knee replacement surgery. Cochrane Database Syst Rev. 2020;12:CD012874.
13. Wall PD, Ahmed I, Metcalfe A, Price AJ, Seers K, Hutchinson CE, et al. Safety and feasibility evaluation of tourniquets for total knee replacement (SAFE-TKR): study protocol. BMJ Open. 2018;8(4):e022067.
14. Bayliss LE, Culliford D, Monk AP, Glyn-Jones S, Prieto-Alhambra D, Judge A, et al. The effect of patient age at intervention on risk of implant revision after total replacement of the hip or knee: a population-based cohort study. Lancet. 2017;389(10077):1424-30.
15. Pijls BG, Valstar ER, Nouta KA, Plevier JW, Fiocco M, Middeldorp S, et al. Early migration of tibial components is associated with late revision: a systematic review and meta-analysis of 21,000 knee arthroplasties. Acta Orthop. 2012;83(6):614-24.
16. Alcelik I, Pollock RD, Sukeik M, Bettany-Saltikov J, Armstrong PM, Fismer P. A comparison of outcomes with and without a tourniquet in total knee arthroplasty: a systematic review and meta-analysis of randomized controlled trials. J Arthroplasty. 2012;27(3):331-40.
17. Ahmed SM, Ahmad R, Case R, Spencer RF. A study of microbial colonisation of orthopaedic tourniquets. Ann R Coll Surg Engl. 2009;91(2):131-4.
18. Berman AT, Parmet JL, Harding SP, Israelite CL, Chandrasekaran K, Horrow JC, et al. Emboli observed with use of transesophageal echocardiography immediately after

For peer review only

For peer review only - http://bmjopen.bmj.com/site/about/guidelines.xhtml

tourniquet release during total knee arthroplasty with cement. The Journal of bone and joint surgery American volume. 1998;80(3):389-96.
19. Sulek CA, Davies LK, Enneking FK, Gearen PA, Lobato EB. Cerebral microembolism diagnosed by transcranial Doppler during total knee arthroplasty: correlation with transesophageal echocardiography. Anesthesiology. 1999;91(3):672-6.
20. Garbedian S, Sternheim A, Backstein D. Wound healing problems in total knee arthroplasty. Orthopedics. 2011;34(9):e516-8.
21. Clarke MT, Longstaff L, Edwards D, Rushton N. Tourniquet-induced wound hypoxia after total knee replacement. J Bone Joint Surg Br. 2001;83(1):40-4.
22. Carden DL, Granger DN. Pathophysiology of ischaemia-reperfusion injury. J Pathol. 2000;190(3):255-66.
23. Namba RS, Cafri G, Khatod M, Inacio MC, Brox TW, Paxton EW. Risk factors for total knee arthroplasty aseptic revision. J Arthroplasty. 2013;28(8 Suppl):122-7.
24. Kerkhoffs GM, Servien E, Dunn W, Dahm D, Bramer JA, Haverkamp D. The influence of obesity on the complication rate and outcome of total knee arthroplasty: a meta-analysis and systematic literature review. J Bone Joint Surg Am. 2012;94(20):1839-44.
25. Sabah SA, Henckel J, Cook E, Whittaker R, Hothi H, Pappas Y, et al. Validation of primary metal-on-metal hip arthroplasties on the National Joint Registry for England, Wales and Northern Ireland using data from the London Implant Retrieval Centre: a study using the NJR dataset. The bone & joint journal. 2015;97-B(1):10-8.
26. Bohm E, Zhu N, Gu J, de Guia N, Linton C, Anderson T, et al. Does adding antibiotics to cement reduce the need for early revision in total knee arthroplasty? Clin Orthop Relat Res. 2014;472(1):162-8.

For peer review only

For peer review only - http://bmjopen.bmj.com/site/about/guidelines.xhtml

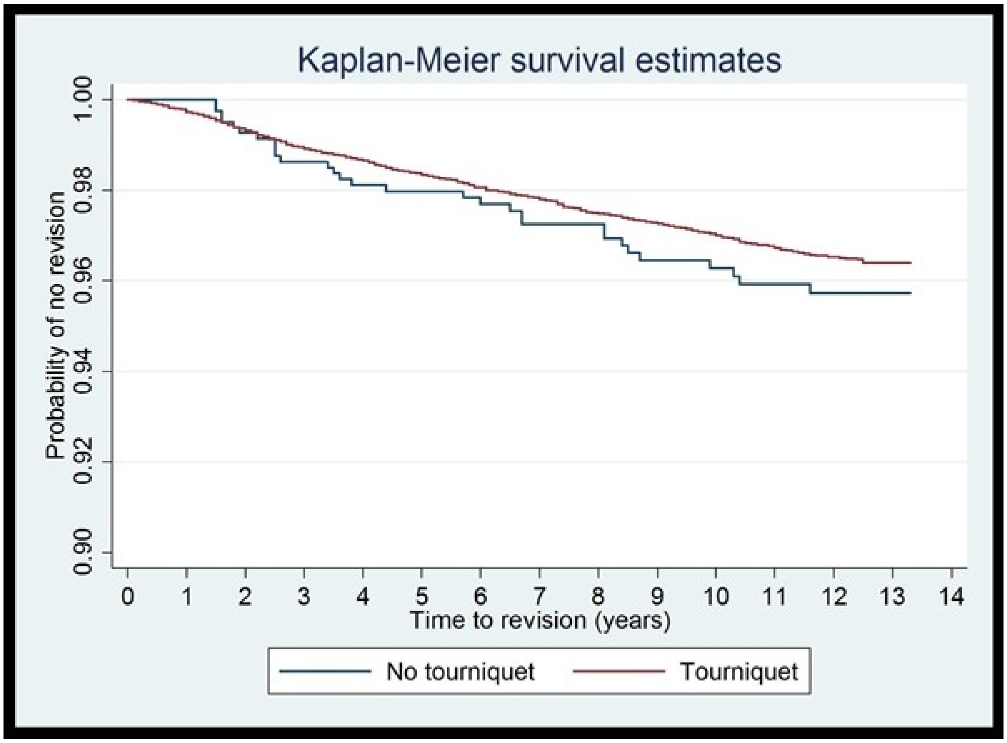

Figure 1: Time to all cause revision by tourniquet use

158x116mm (96 x 96 DPI)

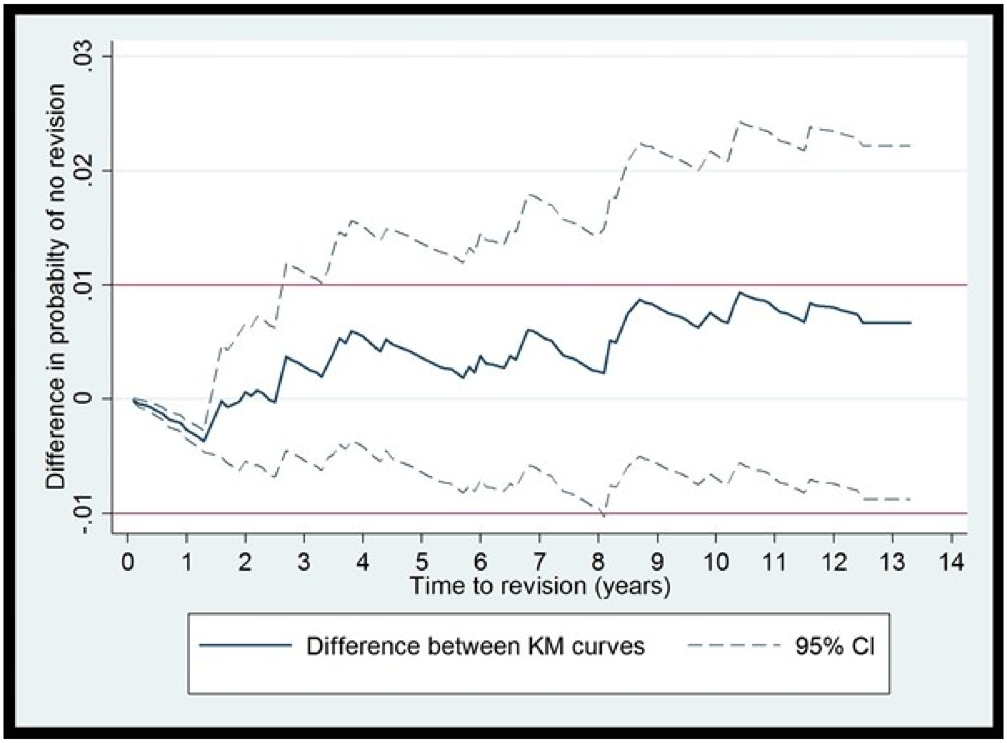

Figure 2: Difference in time to revision (TKR with tourniquet - TKR without tourniquet), over time (unadjusted)

158x116mm (96 x 96 DPI)

STROBE Statement—Checklist of items that should be included in reports of ***cohort studies***

	Item No	Recommendation	Page number
**Title and abstract**	1	(*a*) Indicate the study's design with a commonly used term in the title or the abstract	1
		(*b*) Provide in the abstract an informative and balanced summary of what was done and what was found	2
**Introduction**			
Background/rationale	2	Explain the scientific background and rationale for the investigation being reported	4
Objectives	3	State specific objectives, including any prespecified hypotheses	4
**Methods**			
Study design	4	Present key elements of study design early in the paper	5
Setting	5	Describe the setting, locations, and relevant dates, including periods of recruitment, exposure, follow-up, and data collection	5
Participants	6	(*a*) Give the eligibility criteria, and the sources and methods of selection of participants. Describe methods of follow-up	5
		(*b*) For matched studies, give matching criteria and number of exposed and unexposed	
Variables	7	Clearly define all outcomes, exposures, predictors, potential confounders, and effect modifiers. Give diagnostic criteria, if applicable	5
Data sources/ measurement	8*	For each variable of interest, give sources of data and details of methods of assessment (measurement). Describe comparability of assessment methods if there is more than one group	5
Bias	9	Describe any efforts to address potential sources of bias	5
Study size	10	Explain how the study size was arrived at	5
Quantitative variables	11	Explain how quantitative variables were handled in the analyses. If applicable, describe which groupings were chosen and why	
Statistical methods	12	(*a*) Describe all statistical methods, including those used to control for confounding	5
		(*b*) Describe any methods used to examine subgroups and interactions	
		(*c*) Explain how missing data were addressed	
		(*d*) If applicable, explain how loss to follow-up was addressed	
		(*e*) Describe any sensitivity analyses	
**Results**			
Participants	13*	(a) Report numbers of individuals at each stage of study—eg numbers potentially eligible, examined for eligibility, confirmed eligible, included in the study, completing follow-up, and analysed	6
		(b) Give reasons for non-participation at each stage	
		(c) Consider use of a flow diagram	
Descriptive data	14*	(a) Give characteristics of study participants (eg demographic, clinical, social) and information on exposures and potential confounders	8
		(b) Indicate number of participants with missing data for each variable of interest	
		(c) Summarise follow-up time (eg, average and total amount)	9
Outcome data	15*	Report numbers of outcome events or summary measures over time	9
Main results	16	(*a*) Give unadjusted estimates and, if applicable, confounder-adjusted estimates and their precision (eg, 95% confidence interval). Make clear	11

For peer review only - http://bmjopen.bmj.com/site/about/guidelines.xhtml

		which confounders were adjusted for and why they were included	
		(*b*) Report category boundaries when continuous variables were categorized	
		(*c*) If relevant, consider translating estimates of relative risk into absolute risk for a meaningful time period	
Other analyses	17	Report other analyses done—eg analyses of subgroups and interactions, and sensitivity analyses	
**Discussion**			
Key results	18	Summarise key results with reference to study objectives	12
Limitations	19	Discuss limitations of the study, taking into account sources of potential bias or imprecision. Discuss both direction and magnitude of any potential bias	14
Interpretation	20	Give a cautious overall interpretation of results considering objectives, limitations, multiplicity of analyses, results from similar studies, and other relevant evidence	15
Generalisability	21	Discuss the generalisability (external validity) of the study results	13
**Other information**			
Funding	22	Give the source of funding and the role of the funders for the present study and, if applicable, for the original study on which the present article is based	16

*Give information separately for exposed and unexposed groups.

Note: An Explanation and Elaboration article discusses each checklist item and gives methodological background and published examples of transparent reporting. The STROBE checklist is best used in conjunction with this article (freely available on the Web sites of PLoS Medicine at http://www.plosmedicine.org/, Annals of Internal Medicine at http://www.annals.org/, and Epidemiology at http://www.epidem.com/). Information on the STROBE Initiative is available at http://www.strobe-statement.org.

For peer review only
